# Morphometric measurements of intraoral anatomy in children with Beckwith-Wiedemann syndrome: a novel approach

**DOI:** 10.1186/s13023-024-03350-3

**Published:** 2024-10-17

**Authors:** Dominic J. Romeo, Andrew M. George, Jonathan H. Sussman, Manisha Banala, Andrew Wiemken, Meagan Wu, Jinggang J. Ng, Jesse A. Taylor, Richard J. Schwab, Christopher M. Cielo, Jennifer M. Kalish

**Affiliations:** 1 Division of Plastic, Reconstructive, and Oral Surgery, Children’s Hospital of Philadelphia, 3401 Civic Center Blvd, Philadelphia, PA 19104 USA; 2 Division of Human Genetics, Children’s Hospital of Philadelphia, 3401 Civic Center Blvd, Philadelphia, PA 19104 USA; 3 Division of Sleep Medicine, University of Pennsylvania Perelman School of Medicine, Philadelphia, PA USA; 4 Division of Pulmonary & Sleep Medicine, Children’s Hospital of Philadelphia, 3401 Civic Center Blvd, Philadelphia, PA 19104 USA; 5 Departments of Pediatrics and Genetics, Perelman School of Medicine, University of Pennsylvania, Philadelphia, PA USA

**Keywords:** Morphometrics, Macroglossia, Beckwith-Wiedemann syndrome, Glossectomy

## Abstract

**Background:**

An easy-to-use tool to objectively measure intraoral anatomy with meaningful clinical correlations may improve care for patients with Beckwith-Wiedemann syndrome (BWS), who commonly have symptomatic macroglossia.

**Methods:**

Children aged 2–17 years with BWS were enrolled between 12/2021 and 01/2024. Digital intraoral photographs with a laser ruler were taken, and morphometric measurements were made using ImageJ software. Relationships between morphometrics and outcomes including BWS clinical score, percentage mosaicism, and incidence of tongue reduction surgery were examined using t-tests and multivariate linear models.

**Results:**

Pharyngeal morphometric measurements were obtained in 49 patients with BWS. Mouth area, width, and height differed significantly across BWS molecular subtypes. Right-to-left tongue width and mouth width were larger in those with loss of methylation at imprinting control region 2 (IC2 LOM) than other BWS variants. Patients with paternal uniparental isodisomy of chromosome 11p15 (pUPD11) had narrower mouths than others. Those with tongue reduction surgery had more tongue ridging than those without surgery. There were correlations between mouth area and BWS clinical score, tongue width and BWS clinical score, and tongue length and percentage mosaicism.

**Conclusion:**

Intraoral morphometric measurements are associated with phenotypic burden in BWS. Tongue morphology varies across the BWS spectrum, with IC2 LOM having wider tongues and mouths, and pUPD11 having narrower mouths. Tongue ridging is more common in those selected for surgery. Intraoral morphometric measurements may be safely obtained at low costs across centers caring for children with BWS or others at risk of upper airway obstruction.

**Supplementary Information:**

The online version contains supplementary material available at 10.1186/s13023-024-03350-3.

## Background

Beckwith-Wiedemann syndrome (BWS) is a congenital overgrowth and cancer predisposition disorder caused by (epi)genetic changes on chromosome 11p15 [1–3] that presents with macroglossia in roughly 85% of patients [4]. Pediatric patients with macroglossia may experience difficulties in breathing, speech, feeding, dentoskeletal development, and have an increased risk of obstructive sleep apnea [1, 2, 5–10]. Tongue reduction surgery has been shown to improve these symptoms [11–15]. The decision to perform surgery on these patients is not standardized across centers [10, 16, 17], and more data are needed to better understand macroglossia in BWS.

BWS has been re-classified as a clinical spectrum, as patients may exhibit a range of clinical features [7]. A scoring system was developed to quantify phenotypic severity in BWS [2, 7], under which patients were assigned two points for “cardinal” features such as macroglossia and one point for “suggestive” features such as macrosomia. Confirmatory blood testing is recommended when patients have at least one cardinal feature, and those with scores of four or more including a cardinal feature are clinically diagnosed with BWS [2]. Patients diagnosed with BWS undergo methylation analysis of either blood or an affected tissue [18] that examines imprinting control regions 1 and 2 (IC1 and IC2) on chromosome 11p15, *CDKN1C* gene analysis, and/or copy number analysis or chromosome microarray analysis [2, 7]. Roughly 50% of patients with molecularly confirmed BWS have a loss of methylation at IC2 (IC2 LOM) [7, 19], 5–10% have gain of methylation at IC1 (IC1 GOM), 20% have paternal uniparental isodisomy of chromosome 11p15 (pUPD11), 5% have *CDKN1C* variants, and less than 5% have other chromosomal abnormalities in the 11p15 region [7, 20, 21].

BWS is a mosaic disease, meaning that patients have a mixture of unaffected cells and those with genetic or epigenetic changes [6, 7, 19, 22–26]. Blood mosaicism data can provide useful information about overall disease burden and can be obtained non-invasively then compared across patients with different molecular causes of BWS [18].

A common complication and surgical indication in patients with macroglossia and BWS is obstructive sleep apnea (OSA) [27], which has an estimated prevalence of 48% [28]. Upper airway soft tissue enlargement exacerbates breathing difficulties [29–32], and puts these patients at risk for OSA. When measured on magnetic resonance imaging (MRI) and/or computed tomography (CT), larger tongues and increased pharyngeal soft tissue volume impart an increased risk of developing OSA [30, 31, 33–35]. However, MRI and CT scans are expensive, often require sedation in pediatric populations, and expose patients to radiation [36–39], making them impractical screening tools for pediatric patients with BWS who rarely have indications for cranial imaging.

A low-cost and easy-to-use way to objectively measure the tongue with meaningful clinical correlations would improve risk stratification in patients with BWS. Recently, digital morphometric measurements using a laser ruler have been shown to quantify tongue size, airway visibility, and Mallampati scores in controls and patients with OSA [40]. However, the digital morphometric measurements have not been used to quantify macroglossia severity in pediatric patients or in those with BWS. This study aims to address this gap by assessing correlations between tongue morphometrics and clinical characteristics including BWS clinical score, percentage mosaicism, BWS Index of macroGlossia (BIG) score, sleep apnea, and surgical incidence in patients with BWS. We hypothesize that increased morphometric measurements are associated with more severe clinical outcomes in patients with BWS.

## Methods

This was a single-center cohort study. Institutional review board approval (IRB 13-010658) and consent from each patient were obtained. Children with Beckwith-Wiedemann syndrome able to sit upright who were seen by the team geneticist (JMK) for regular clinical visits were enrolled in this study. Demographic variables and history of tongue reduction surgery were recorded. Blood methylation testing was reviewed, and the BWS molecular subtypes which were recorded as either 11p15 duplication, *CDKN1C* variant, genome-wide paternal uniparental isodisomy (GWpUPD), IC1 GOM, IC2 LOM, and pUPD11. Patients with a clinical diagnosis of BWS were labeled accordingly. A BWS clinical score was calculated according to the International Consensus Scoring system [7]. Blood mosaicism percentage was calculated using established methodology (Supplementary Table 1) in eligible patients [18, 41]. Beckwith-Wiedemann syndrome Index of macroGlossia (BIG) scores were recorded in both the pre- and post-surgical cohorts. Polysomnography variables included evaluation age, apnea-hypopnea index (AHI), and oxyhemoglobin saturation (SpO_2_) nadir. Polysomnographic data that were obtained prior to tongue reduction surgery were excluded.

Previously, our team analyzed 459 patients with BWS and developed a macroglossia severity scoring system, the BWS Index of macroGlossia (BIG) [42]. Patients were classified from BIG0 to BIG3, where BIG0 included those without macroglossia; BIG1 included those with macroglossia not protruding beyond the teeth/alveolus; BIG2 included those with tongue protrusion past the teeth/alveolus to the lips but that can be contained within the mouth; and BIG3 included those with tongues that protrude beyond the teeth/alveolus and lips but that cannot be closed within the mouth. BIG score was found to have significant correlations with phenotypic severity and tongue reduction surgical incidence.

**Fig. 1:**
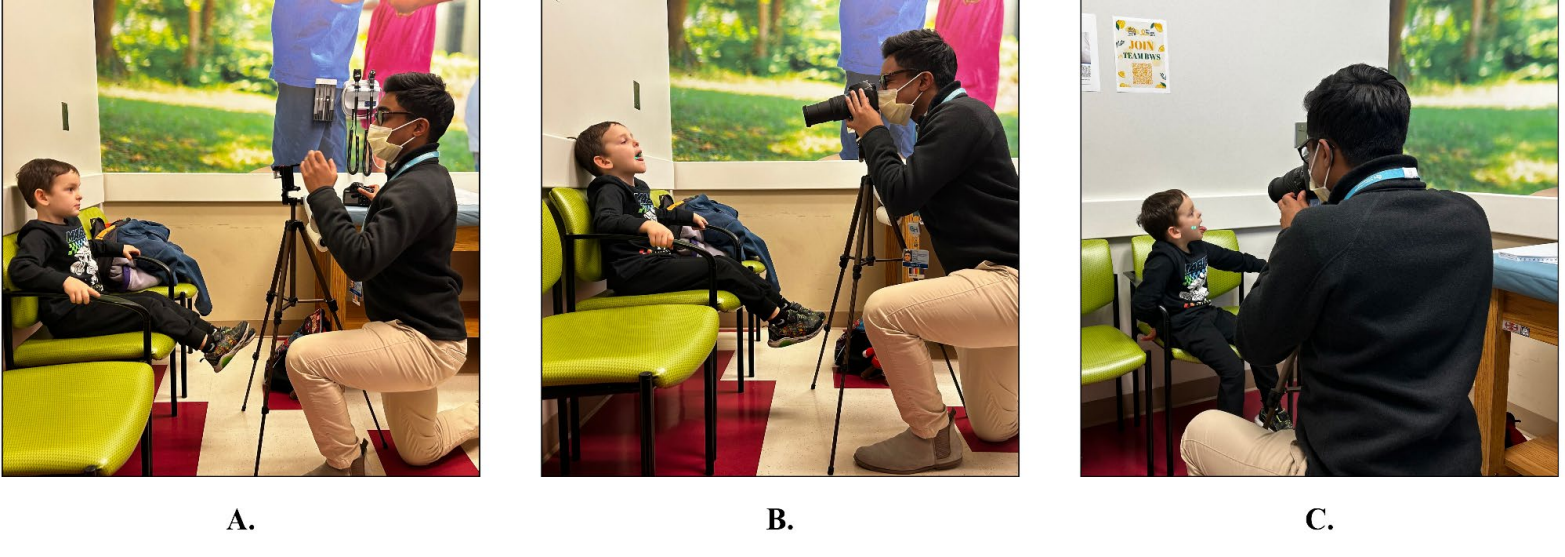
Depiction of how to obtain morphometric images using a camera and an intraoral laser composed of a right-angle beam splitter and mirror. (**A**) Shows a patient seated with his head in a neutral position and his line of site parallel to the floor; (**B**) depicts the camera and laser ruler roughly 1 m from patient capturing open mouth no phonation photographs; and (**C**) shows the capturing of photographs in the tongue extended maximally lateral position

To obtain photographs for morphometric measures, each subject was seated with their head in a neutral position and line of sight parallel to the floor, a method previously used and shown to have meaningful clinical correlations [40, 43]. A camera and laser ruler were positioned roughly one meter from subjects who were instructed to open their mouths. Images were obtained using a Canon Rebel EOS T3 digital camera and an intraoral laser composed of a right angle beam splitter and mirror that were aligned such that the two parallel beams projected forward 1 cm apart, as has been described (Fig. 1) [40]. A camera was attached to the laser ruler and digital photographs were taken to capture the laser beams near anatomical regions of interest for further study. The distance between the lasers was used to calculate quantitative measures from the photographs.

**Fig. 2:**
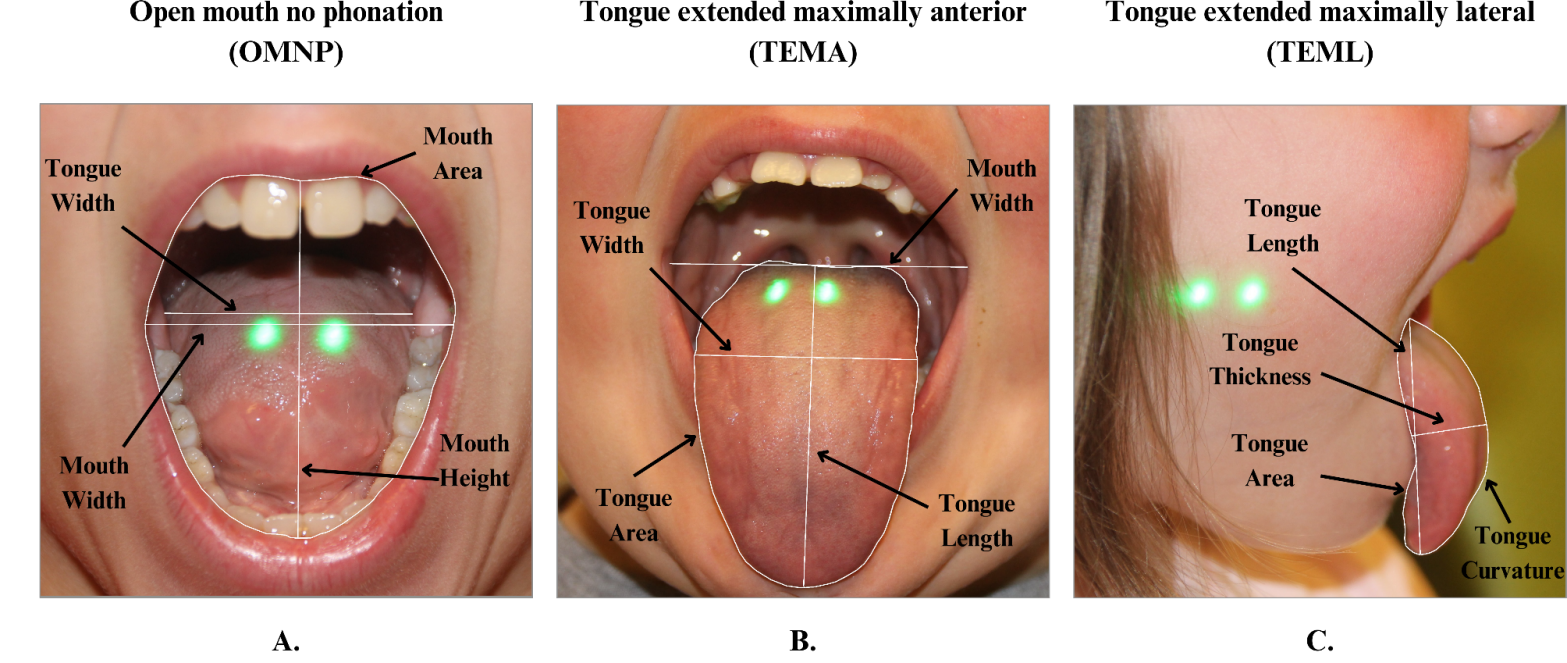
Example photographs depicting morphometric measurements with (**A**) showing OMNP measurements of tongue width, mouth width, mouth height, and mouth area; (**B**) showing TEMA measurements of the tongue width, tongue length, tongue area, and mouth width; and (**C**) showing TEML measurement of tongue length, tongue thickness, tongue curvature, and tongue area

**Fig. 3:**
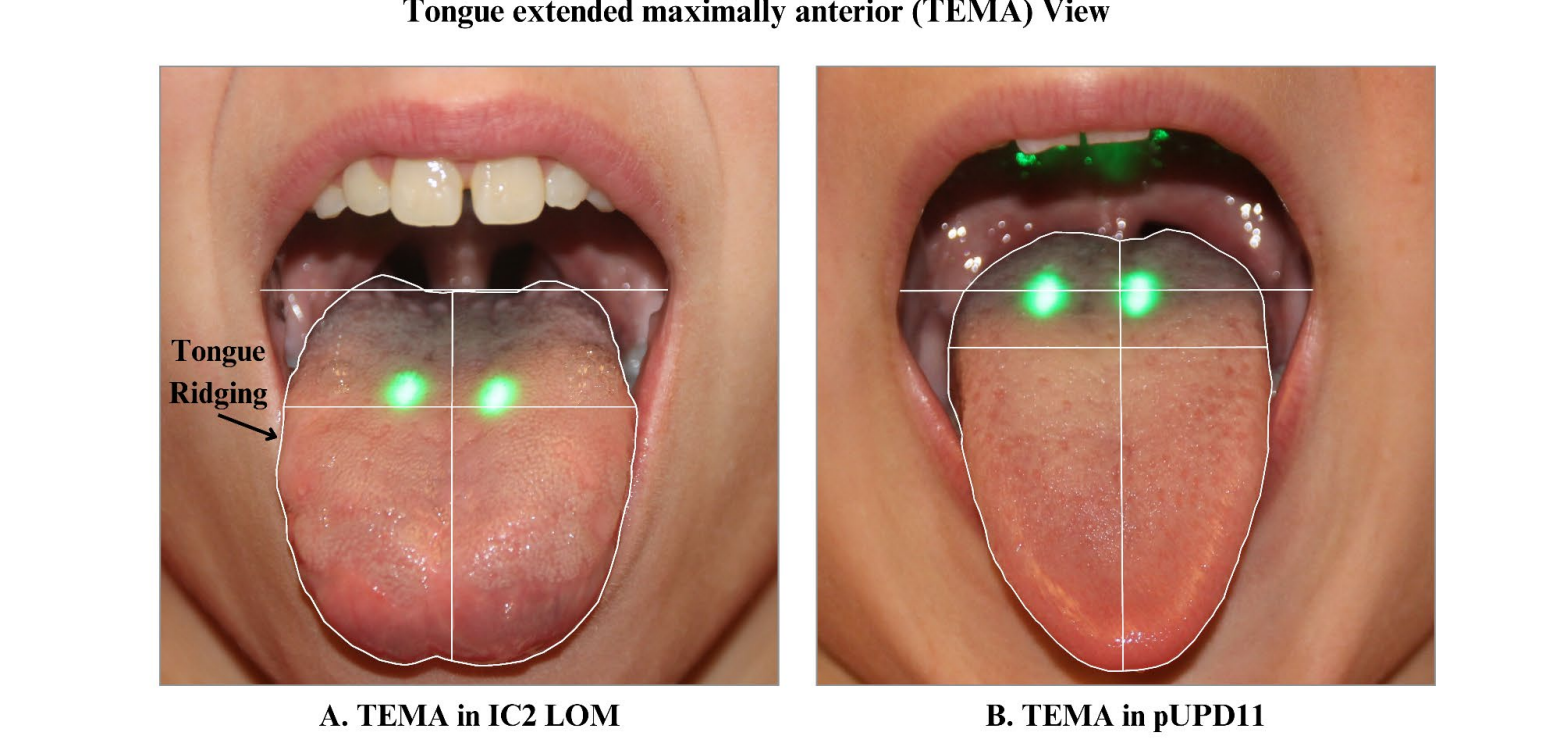
Comparison of TEMA photographs demonstrating a wider tongue and mouth in the patient with IC2 LOM (**A**) and a narrower mouth in the patient with pUPD11 (**B**). The patient with IC2 LOM also has significant tongue ridging, which was more common in those who had surgery compared to those who did not

Three intraoral photographs were taken, two anteriorly and one laterally. The two anterior positions included open mouth no phonation (OMNP), tongue extended maximally anterior (TEMA), and tongue extended maximally lateral (TEML). Regarding OMNP, the distance between lasers (scale), mouth area (MArea), mouth width (MWidth), mouth height (MHeight), and tongue width (TWidth) were measured. For TEMA, distance between lasers (scale), mouth width (MWidth), tongue area (TArea), tongue width (TWidth), and tongue length (TLength) were measured. Regarding TEML, distance between laser (scale), tongue area (TArea), tongue curvature (TCurve), tongue length (TLength), and tongue thickness (TThick) were measured (Fig. 2). Categorical measures of pharyngeal airway visibility, Mallampati scores, tongue ridging, tongue curvature, tonsil hypertrophy grade, and pharyngeal narrowing were also obtained. Morphometric measurements were calculated using ImageJ software (Fig. 3) [44].

Relationships among morphometric measurements, BWS clinical score, level of affected cells in the blood, BIG score, polysomnography findings, and surgical incidence were examined using R v4.3.2. Continuous variables were reported as median (interquartile range) or mean ± SD and compared using Student’s t-tests or analysis of variance analysis. Categorical variables were compared using Chi-square tests. Pearson’s correlation and multivariate linear models were utilized to analyze associations between continuous variables. Multivariate logistic regression was used to model risk of surgery. Statistical tests used for each comparison are indicated in the corresponding tables and figures. The Benjamini-Hochberg method was applied to correct for multiple hypothesis testing within each table with a false discovery rate of < 0.25. All categorical and continuous variables were independently measured by AMG and MB, and the level of agreement between their respective measurements was analyzed.

## Results

Forty-nine children with Beckwith-Wiedemann syndrome underwent the morphometric measurements. All patients (100%) had at least one photograph able to be analyzed on ImageJ. The cohort was comprised of 25 (51%) males and 24 (49%) females with a median (interquartile range) age of 6.13 (4.66–7.85) years. BWS diagnoses were: 1 (1.9%) 11p15 duplication, 2 (3.7%) *CDKN1C* variants, 3 (5.6%) GWpUPD, 3 (5.6%) IC1 GOM, 21 (38.9%) IC2 LOM, 15 (27.8%) pUPD11, and 9 (16.7%) clinical diagnoses (Table 1). The median BWS clinical score was 8 (2–10) and the median blood mosaicism percentage was 57% (51–64%). Regarding BWS Index of macroGlossia scores (BIG), 13 (36.1%) patients were assigned a BIG0, 10 (27.8%) were BIG1, 12 (33.3%) were BIG2, and 1 (2.8%) was BIG3. The AHI was 3.9 events/hour (1.8–7.0) and SpO_2_ nadir was 86% (85–90). 19 patients (36.5%) underwent tongue reduction surgery at a median age of 1.54 (0.82–0.90) years old. The time range between surgery and morphometric measurements was 5.28 (3.27–8.06) years. All categorical and quantitative morphometric measurements were recorded (Table 1).

**Table 1:** Demographic and morphometric measurement overview

Category	Quantification	Category	Quantification	Category	Quantification
Patients	49	**Grade Ridging (***n* = **53)**		**TEMA Tongue Visibility (***n* = **53)**	
Morphometric Measurements	54	0	16 (30.2%)	1	32 (60.4%)
Sex		1	29 (54.7%)	2	1 (1.9%)
Male	25 (51.0%)	2	6 (11.3%)	3	19 (35.8%)
Female	24 (49.0%)	3	2 (3.8%)	4	1 (1.9%)
Age (years)	6.13 (4.66–7.85)	**Tonsil Grade (***n* = **16)**		**TEMA Laser Quality (***n* = **53)**	
BMI (kg/m^2^)	17.74 (16.02–18.96)	0	6 (37.5%)	1	52 (98.1%)
BWS Diagnosis (*n* = 54)		1	2 (12.5%)	3	1 (1.9%)
11p15 Duplication	1 (1.9%)	2	3 (18.8%)	**TEML Tongue Quality (***n* = **29)**	
CDKN1C	2 (3.7%)	3	5 (31.2%)	1	28 (96.6%)
GWpUPD	3 (5.6%)	**Lateral Wall Grade (***n* = **16)**		2	1 (3.4%)
IC1 GOM	3 (5.6%)	1	1 (6.2%)	**TEML Tongue Angle (***n* = **29)**	
IC2 LOM	21 (38.9%)	2	12 (75.0%)	1	22 (75.9%)
pUPD11	15 (27.8%)	3	3 (18.8%)	2	1 (3.4%)
Clinical	9 (16.7%)	**OMNP Mallampati Score (***n* = **8)**		3	6 (20.7%)
BWS Clinical Score (*n* = 49)	8.00 (2.00–10.00)	1	2 (25.0%)	**TEML Laser Quality (***n* = **29)**	
Percent Mosaicism (*n* = 21)	57% (51–64%)	2	5 (62.5%)	1	29 (100.0%)
BIG Score		3	1 (12.5%)	**OMNP**	
0	13 (36.1%)	**OMNP Tongue Visibility (***n* = **42)**		Scale (*n* = 42)	290.7 (253.9–330)
1	10 (27.8%)	1	1 (2.4%)	MArea (*n* = 34)	1,084,000 (820300–1355000)
2	12 (33.3%)	2	1 (2.4%)	MWidth (*n* = 40)	1205 (1058–1430)
3	1 (2.8%)	3	27 (64.3%)	MHeight (*n* = 35)	1138 (945.9–1305)
Polysomnography (*n* = 18)		4	13 (31.0%)	TWidth (*n* = 27)	1110 (934.3–1232)
AHI (*n* = 18)	3.85 (1.82–7.04)	**OMNP Laser Quality (***n* = **42)**		**TEMA**	
SpO2 (*n* = 18)	0.86 (0.85–0.90)	1	42 (100.0%)	Scale (*n* = 53)	277 (236.3–319)
Prior Glossectomy		**TEMA Mallampati Score (***n* = **16)**		MWidth (*n* = 48)	1256 (1085–1344)
Yes	19 (36.5%)	1	1 (6.2%)	TArea (*n* = 33)	1,075,000 (803700–1443000)
Age (years; *n* = 16)	1.54 (0.82–2.48)	2	13 (81.2%)	TWidth (*n* = 53)	1072 (894–1210)
No	33 (63.5%)	3	2 (12.5%)	TLength (*n* = 32)	1214 (1009–1414)
		**TEMA Tongue Quality (***n* = **52)**		**TEML**	
		1	46 (88.5%)	Scale (*n* = 29)	259 (233.1–309)
		2	3 (5.8%)	TArea (*n* = 28)	452,300 (376900–590600)
		3	1 (1.9%)	TCurve (*n* = 27)	1564 (1317–1686)
		4	2 (3.8%)	TLength (*n* = 27)	1158 (1045–1295)
				TThick (*n* = 28)	478.8 (437.3–608.5)

Values are reported as median (interquartile range)

BMI: body mass index, BWS: Beckwith-Wiedemann Syndrome, BIG: BWS Index of macroGlossia, AHI: apnea-hypopnea index, SpO_2_ nadir: oxygen saturation nadir, OMNP: open mouth no phonation, TEMA: tongue extended maximally anterior, TEML: tongue extended maximally lateral

**Fig. 4:**
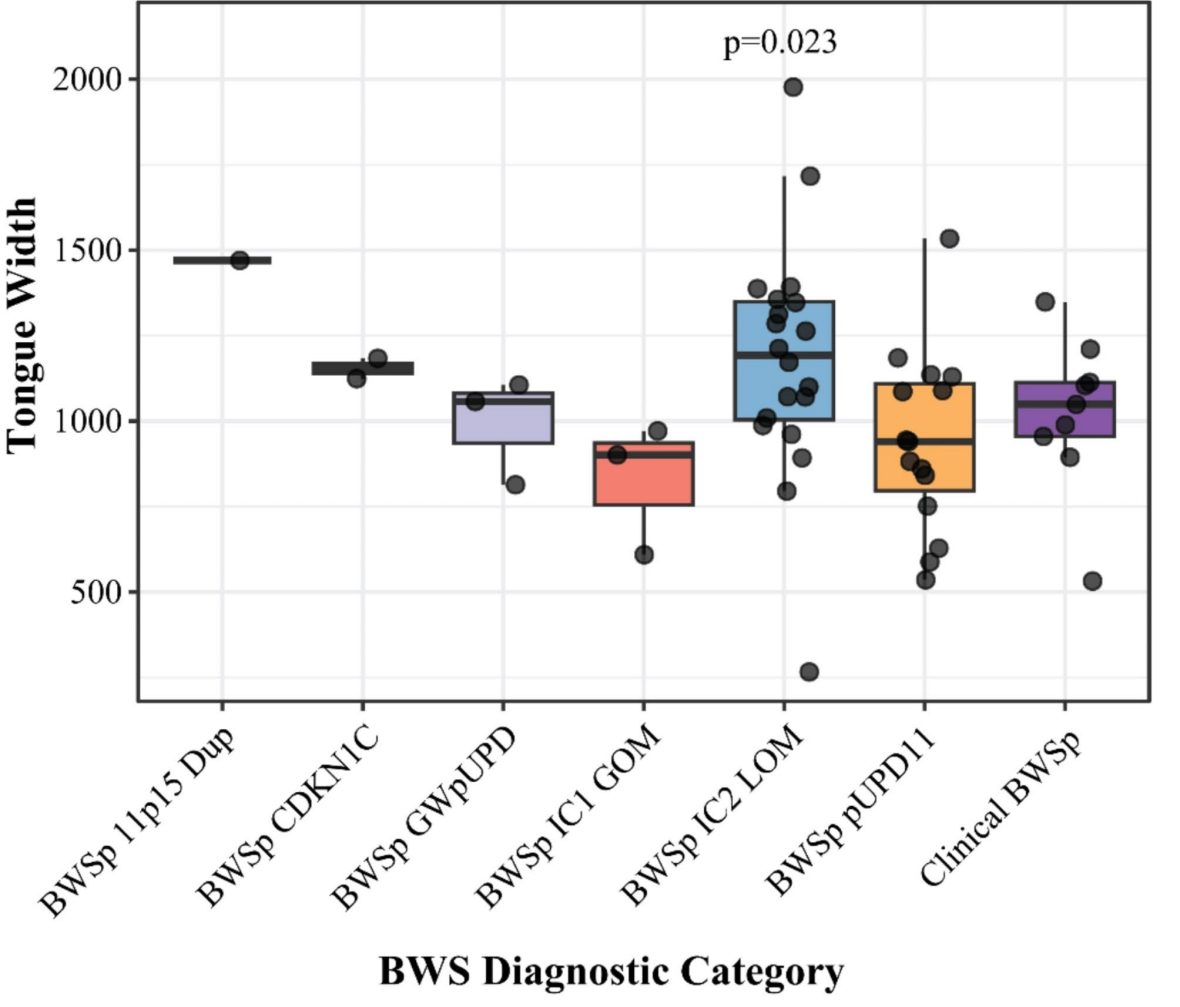
Tongue width on tongue extended maximally anterior view; p-values correspond to that diagnosis compared to all others on a two-sided Student’s t-test

**Fig. 5:**
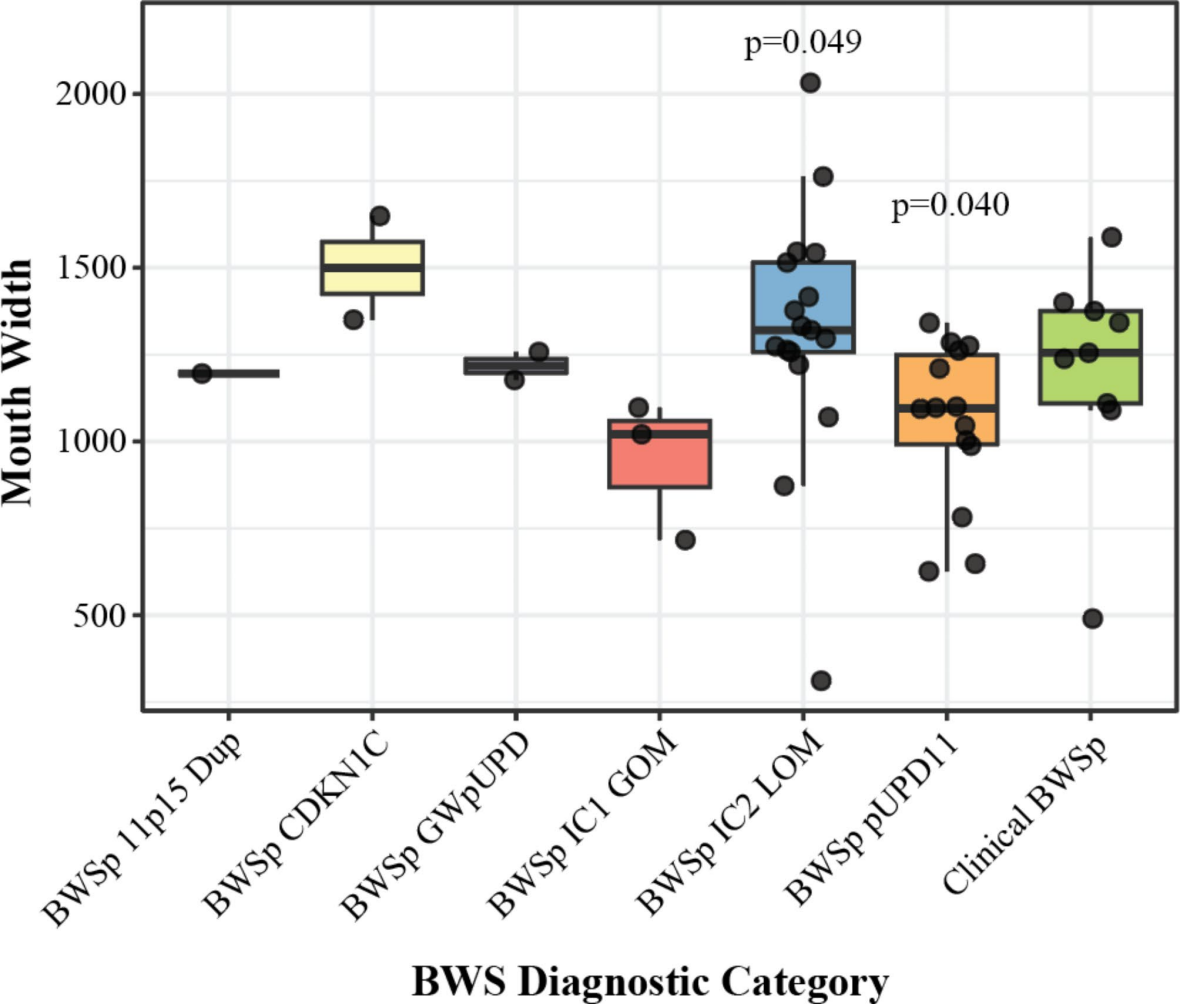
Mouth width on tongue extended maximally anterior view; p-values correspond to that diagnosis compared to all others on a two-sided Student’s t-test

Examining morphometric measurements showed that in the open mouth no phonation photographs (OMNP), MArea, MWidth, and MHeight differed significantly across the various molecular diagnoses (Table 2). Similar trends were observed in tongue and mouth width in the TEMA view, though significance was not achieved. Further subgroup analysis revealed that in the TEMA view, tongue width (*p* = 0.023; Fig. 4) and mouth width (*p* = 0.049; Fig. 5) were significantly larger in the IC2 LOM cohort than the remaining patients. Additionally, those with pUPD11 had significantly narrower mouths than others (*p* = 0.040).

**Table 2:** Associations between molecular diagnoses and various clinical outcomes

	IC1 GOM (*n* = 3)	IC2 LOM (*n* = 21)	pUPD11 (*n* = 15)	CDKN1C (*n* = 2)	GWpUPD (*n* = 3)	11p15 Duplication (*n* = 1)	Clinical Diagnosis (*n* = 9)	*p*
BWS Clinical Score	3.333 ± 5.774	8.333 ± 3.23	5.80 ± 4.99	4.00 ± 5.66	2.33 ± 4.04	0	7.00 ± 0.82	0.7
Percent Mosaicism	55 ± 1%	55 ± 17%	66 ± 7%	-	-	-	-	*0.137*
BIG Score	0 ± 0	1.769 ± 0.5991	1.00 ± 0.94	1	0.33 ± 0.58	1		*0.004
AHI	10.9	4.79 ± 5.34	5.63 ± 4.34	3.9	-	-	4.05 ± 1.74	*0.759*
SpO_2_	0.79	0.84 ± 0.06	0.87 ± 0.06	0.95	-	-	0.88 ± 0.03	*0.296*
Ridging Grade	1 ± 1	1.19 ± 0.8729	0.79 ± 0.43	0.00 ± 0.00	1.00 ± 1.00	1	0.44 ± 0.53	0.334
Tonsil Grade	-	1.111 ± 1.269	1.67 ± 1.53	3	0	2	3	0.645
Lateral Wall Grade	2	2.25 ± 0.4629	2.00 ± 0.00	1.50 ± 0.71	-	3	2	0.206
OMNP								
Mallampati	-	1.8 ± 0.8367	2.00	2	-	2	-	0.824
Scale	263.53 ± 21.92	277.35 ± 75.18	303.9 ± 64.34	301.8 ± 31.46	357.8 ± 13.01	327.03	256.9 ± 66.86	*0.566*
MArea	9.285E + 5 ± 2.628E + 5	1.0400E + 6 ± 5.522E + 5	1.055E + 6 ± 4.289E + 5	8.388E + 5	8.187E + 6 ± 9.823E + 6	2.999E + 7	9.035E + 6 ± 4.594E + 6	*<0.0001
MWidth	1128 ± 56.82	1219 ± 369.9	1226 ± 266.0	1070	1526 ± 398.5	2740	896.9 ± 538.0	*0.00059
MHeight	1030 ± 276.8	1152 ± 477.3	1114 ± 246.0	1384 ± 524.3	1339	2918	971.8 ± 337.4	*0.0112
TWidth	934.3 ± 46.27	1009 ± 403.9	1113 ± 299.8	1102 ± 170.7	1363 ± 328.1	1627	953.6 ± 288.6	*0.518*
TEMA								
Mallampati	2 ± 0	2 ± 0.5774	2.33 ± 0.58	2	-	2	2.00 ± 0.00	0.977
Scale	228.7 ± 75.79	290.4 ± 86.73	245.0 ± 58.05	330.7 ± 34.90	258.2 ± 53.06	201.68	278.7 ± 62.30	*0.35*
MWidth	944.7 ± 201.6	1318 ± 366.9	1054 ± 229.7	1499 ± 211.2	1217 ± 56.63	1195	1210 ± 310.0	*0.154*
TArea	1.085E + 6 ± 2.407E + 4	1.072E + 6 ± 5.504E + 6	1.109E + 6 ± 5.282E + 5	1.397E + 6 ± 1.578E + 5	8.281E + 5 ± 1.355E + 5	9.750E + 5	1.194E + 06 ± 4.861E + 05	*0.947*
TWidth	827.2 ± 192.0	1179 ± 349.1	942.0 ± 263.3	1154 ± 42.12	992.3 ± 156.2	1470	1022 ± 229.1	*0.135*
TLength	1324 ± 110.8	1088 ± 380.6	1269 ± 391.8	1446 ± 218.3	1104	1298	1309 ± 398.8	*0.835*
TEML								
Scale	258.6 ± 0.56	267.4 ± 59.36	255.6 ± 59.36	281.0 ± 39.6	311.1	204.5	274.3 ± 53.99	*0.884*
TArea	4.035E + 5 ± 8.446E + 4	4.610E + 5 ± 2.220E + 5	4.896E + 5 ± 1.704E + 5	4.841E + 5 ± 1.519E + 5	3.986E + 5	5.110E + 5	7.632E + 5 ± 3.007E + 5	*0.481*
TCurve	1564	1365 ± 326.8	1560 ± 340	1542 ± 446	1422	1844	1965 ± 367.9	*0.236*
TLength	1190 ± 68.54	1063 ± 389.0	1199 ± 179.8	1340 ± 343.5	1184	1466	1423 ± 270.5	*0.588*
TThick	407.6 ± 57.44	532.0 ± 161.1	496.0 ± 80.99	442.2 ± 2.044	440.4	427.8	652.7 ± 158.5	*0.472*

BMI: body mass index, BWS: Beckwith-Wiedemann Syndrome, BIG: BWS Index of macroGlossia, AHI: apnea-hypopnea index, SpO_2_ nadir: oxygen saturation nadir, OMNP: open mouth no phonation, TEMA: tongue extended maximally anterior, TEML: tongue extended maximally lateral

Values are reported as mean ± standard deviation

*p-values corresponding to analysis of variance analysis are noted in italicized text*

p-values corresponding to chi-squared tests are noted in underlined text

*indicates p-values that remain statistically significant following Benjamini-Hochberg correction with a false discovery rate of < 0.25

Comparing those who had tongue reduction surgery to those who did not, revealed significantly more ridging in individuals with surgery (1.37 ± 0.76 vs. 0.62 ± 0.61). There was no difference in any morphometric measurement between those who had surgery in the last five years and those who had it longer than five years ago. Additionally, tongues of those who had surgery had significantly less curvature than those in patients who did not have surgery. Patients who underwent tongue reduction surgery also had significantly higher BIG scores (1.86 ± 0.53 vs. 0.50 ± 0.67) and BWS clinical scores (9.21 ± 2.80 vs. 4.79 ± 4.31) than those who did not (Table 3).

**Table 3:** Outcome variance between surgery and non-surgery cohorts

	History of Tongue Reduction Surgery		
	**No**	**Yes**	***p***
BWS Clinical Score	4.79 ± 4.31	9.21 ± 2.80	*0.009
Percent Mosaicism	64 ± 8%	57 ± 15%	*0.210*
BIG Score	0.50 ± 0.67	1.86 ± 0.53	*<0.001
AHI	6.59 ± 3.80	4.07 ± 4.62	*0.232*
SpO_2_	0.87 ± 0.05	0.85 ± 0.06	*0.411*
Ridging Grade	0.62 ± 0.61	1.37 ± 0.76	*0.008
Tonsil Grade	1.78 ± 1.39	1.00 ± 1.15	0.268
Lateral Wall Grade	2.00 ± 0.47	2.33 ± 0.52	0.411
OMNP			
Mallampati	1.75 ± 0.50	2.00 ± 0.82	0.549
Scale	288.2 ± 57.97	278.7 ± 82.94	*0.674*
MArea	3.120E + 6 ± 7.081E + 6	1.097E + 6 ± 5.831E + 5	*0.315*
MWidth	1242 ± 494.8	1208 ± 386	*0.816*
MHeight	1162 ± 506.3	1212 ± 494.8	*0.775*
TWidth	1064 ± 319.7	1064 ± 397.4	*1*
TEMA			
Mallampati	2.10 ± 0.32	2.00 ± 0.63	0.359
Scale	272.2 ± 62.8	267.2 ± 93.48	*0.82*
MWidth	1190 ± 275.8	1213 ± 403.9	*0.817*
TArea	1.090E + 6 ± 3.907E + 5	1.154E + 6 ± 7.253E + 5	*0.754*
TWidth	1018 ± 235.6	1141 ± 399.4	*0.175*
TLength	1255 ± 330.3	1131 ± 469.7	*0.431*
TEML			
Scale	270.7 ± 43.09	251.2 ± 62.09	*0.335*
TArea	5.252E + 5 ± 1.470E + 5	4.104E + 5 ± 1.983E + 5	*0.096*
TCurve	1621 ± 259	1320 ± 320	*0.014
TLength	1232 ± 168	1062 ± 407.8	*0.144*
TThick	518.6 ± 125.1	485.6 ± 131.3	*0.511*

AHI: apnea-hypopnea index, SpO_2_: oxygen saturation nadir, BWS: Beckwith-Wiedemann Syndrome, BIG: BWS Index of macroGlossia, OMNP: open mouth no phonation, TEMA: tongue extended maximally anterior, TEML: tongue extended maximally lateral

All values reported as mean ± standard deviation

*p-values corresponding to two-sampled student’s t-tests are noted in italicized text.*

p-values corresponding to chi-squared tests are noted in underlined text.

*indicates p-values that remain statistically significant following Benjamini-Hochberg correction with a false discovery rate of < 0.25

Correlation analysis showed significant correlations between OMNP mouth area and BWS clinical score. Additionally, there were significant correlations between TEMA tongue width and BWS clinical score, TEMA tongue length and percentage mosaicism, as well as between TEML tongue curvature and percentage mosaicism (Table 4). Between the two independent image raters (AMG and MB) assessing categorical variables in photographs, there was a high degree of accuracy (64%) that was significantly greater than random chance (*p* = 0.007). For continuous variables, a Pearson’s correlation also revealed a high agreement between raters (*p* < 0.001).

**Table 4:** Linear models controlling for age at morphometric measurements and body mass index

	AHI		SpO2		Percent Mosaicism		BIG Score			BWS Clinical Score	
	**Coefficient**	**p**	**Coefficient**	**p**	**Coefficient**	**p**	**Coefficient**		**p**	**Coefficient**	**p**
BIG Score	3.78E + 00		-6.07E-02	0.230	-3.09E-02	0.569	-		-	4.15E + 00	*<0.0001
AHI	-	-	-9.92E-03	*0.003	3.08E-02	*0.039	4.91E-02		0.286	2.10E-01	0.256
SpO_2_	-4.74E + 01	*0.003	-	-	-1.94E + 00	0.163	-3.78E + 00		0.230	-2.42E + 01	*0.047
Percent Mosaicism	2.25E + 01	0.039	-2.18E-01	0.163	-	-	-1.09E + 00		0.569	-6.55E + 00	0.505
BWS Clinical Score	4.34E-01	0.256	-1.05E-02	0.047	-4.06E-03	0.505	1.43E-01		*<0.0001	-	-
OMNP											
MArea	-4.30E-07	0.868	2.29E-08	0.618	-2.10E-08	0.765	-1.89E-07		0.100	-3.74E-07	*0.046
MWidth	-7.68E-04	0.879	1.91E-05	0.784	-3.41E-05	0.811	5.60E-04		0.300	-4.84E-04	0.820
MHeight	1.29E-03	0.749	-3.23E-05	0.587	-5.11E-05	0.676	6.78E-04		0.173	-5.72E-04	0.738
TWidth	-7.97E-04	0.849	-2.64E-05	0.754	-8.12E-05	0.490	3.41E-04		0.723	1.24E-03	0.648
TEMA											
TArea	-3.69E-06	0.359	-3.56E-08	0.641	7.85E-08	0.236	2.01E-07		0.581	1.70E-06	0.361
TWidth	2.07E-03	0.618	-1.05E-04	0.076	-2.01E-05	0.822	6.82E-04		0.175	4.36E-03	*0.040
TLength	-5.20E-03	0.208	-4.31E-06	0.958	1.89E-04	*0.012	-1.57E-04		0.742	2.38E-04	0.921
MWidth	-9.42E-04	0.873	-9.26E-05	0.293	-3.64E-05	0.736	3.04E-04		0.527	2.07E-03	0.345
TEML											
TArea	1.03E-05	0.241	9.69E-08	0.448	5.73E-07	0.140	-3.00E-07		0.812	-9.85E-07	0.810
TCurve	5.05E-03	0.273	4.04E-05	0.538	4.20E-04	*0.045	-6.83E-04		0.324	-2.06E-03	0.403
TLength	4.48E-03	0.327	1.88E-05	0.777	9.54E-05	0.560	-3.28E-04		0.645	-2.47E-03	0.339
TThick	2.51E-02	0.099	2.83E-05	0.904	9.80E-04	0.088	5.13E-04		0.812	4.44E-03	0.483

AHI: apnea-hypopnea index, SpO_2_: oxygen saturation nadir, BWS: Beckwith-Wiedemann Syndrome, BIG: BWS Index of macroGlossia, OMNP: open mouth no phonation, TEMA: tongue extended maximally anterior, TEML: tongue extended maximally lateral

*indicates p-values that remain statistically significant following Benjamini-Hochberg correction with a false discovery rate of < 0.25

## Discussion

Patients with Beckwith-Wiedemann syndrome commonly present with macroglossia, but accurately quantifying macroglossia severity remains challenging. While the BWS Index of macroGlossia has added nuance to discussions about macroglossia and has shown correlation with various clinical outcomes [42], objective methods for measuring macroglossia severity remain limited beyond polysomnography. Our study demonstrates that a morphometrics protocol using a laser and digital camera offers a practical and clinically-relevant approach to obtaining quantitative tongue measurements and objectively assessing macroglossia severity. The findings presented here add distinctions to emerging discussions about the spectrum of intraoral morphology across the different BWS molecular diagnoses. Finally, this study highlights that characteristics such as tongue ridging are more common in patients who had tongue reduction surgery than in those who did not.

Many studies have examined associations between intraoral anatomy and clinical outcomes, but these often require expensive equipment, use calibers which are difficult to maneuver in the mouth, are inaccurate, or exclude pediatric populations [40, 43, 45–50]. It is important to characterize soft tissue morphology and risk factors in children predisposed to diseases such as those with BWS or OSA. Still, objective soft tissue data are classically obtained via CT or MRI, exposing children to radiation or requiring sedation, and are therefore usually not obtained [36–39]. In their landmark study of 860 patients (542 with OSA and 318 controls), Schwab et al. demonstrated that tongue morphometric measurements using a digital camera and laser ruler provided accurate and reproducible measurements of the tongue and mouth that were significantly associated with polysomnographic outcomes [40]. However, this previous study did not include children or patients with BWS, limiting its generalizability. Our study builds on this previous work by showing that tongue and mouth measurements are significantly correlated by genetic subtype and with measures of phenotypic severity in BWS including clinical score and percentage mosaicism.

This information is useful because while BWS clinical scores allow objective assessment of phenotypic burden, obtaining this information requires comprehensive patient assessment. Further, the BWS clinical scoring system views macroglossia as a dichotomous variable that is either present or absent, failing to account for the range of macroglossia across patients with BWS [7]. Additionally, while the BIG scores proposed by our group correlate with clinical outcomes such as surgical incidence [42], this system did not involve objective measurements of the oral anatomy and may be challenging to reproduce across clinical settings. The morphometric findings presented here reveal correlations between oral measurements, BWS clinical scores, and BIG scores, offering a quick and reproducible technique to quantify macroglossia severity in patients with BWS.

Obtaining morphometric measurements is inexpensive, does not require sedation, and can be performed in most clinical settings. Children are advised to sit down with their tongues extended for a few seconds while photographs are taken. Images from the three positions are captured, and the entire process takes less than five minutes to complete. As we obtained these images, we found that children as young as two years of age can sit long enough for accurate photographs to be taken. Previously, obtaining tongue morphometric measurements had been shown to be feasible in adults, but not in children. Our study demonstrates that this modality can enhance care not only for children with BWS, but also for those with macroglossia from conditions including Simpson-Golabi-Behmel syndrome [51] and, more broadly, for all patients at risk of upper airway obstruction, such as in children with Down Syndrome or obesity [52, 53].

Assessing these photographs in patients with BWS, we found different morphological features across the various molecular diagnoses. It is well established that the incidence of certain clinical features is higher in some BWS molecular subtypes than other. For instance, patients with IC2 LOM more commonly present with omphalocele, patients with pUPD11 more often have lateralized overgrowth, and most of the patients with IC2 LOM have macroglossia [6]. There is little published literature reporting measurable differences in tongue morphology across those with different BWS molecular subtypes. Our study shows that there are objective differences between the intraoral anatomical measurements between the different BWS molecular subtypes.

Anecdotally, we have observed that those with IC2 LOM tend to have wide and long tongues, and patients with pUPD11 appear to have thick and wide tongues. Testing this hypothesis, we observed that those with IC2 LOM had wider tongues and mouths than others. Interestingly, we did not find a difference between the tongue width or thickness in patients with pUPD11 compared to others with BWS, though we found that they had smaller mouths than others. A recent study from our center assessed a large cohort of patients who underwent tongue reduction surgery, finding that those with pUPD11 made up a large proportion of patients selected for surgery, and that they more commonly had repeat surgery than patients with other BWS subtypes [14]. We initially speculated that a reason for these findings could be larger tongues in those with pUPD11, but morphometrics data demonstrated that patients with pUPD11 did not have larger tongues in the cohort presented here. A possible explanation for the discrepancy between our expectations and measurements is that we were underpowered to make statistically significant conclusions in this patient population using morphometrics. Another possibility is that tongue morphology alone is only one of many factors contributing to the need for surgery, and that the entire clinical picture of patients is needed to determine if a patient requires surgical intervention.

At our center, all patients with Beckwith-Wiedemann syndrome are seen by the team geneticist (JMK) who orders molecular testing and assigns both a BWS clinical score and BIG score. As part of our macroglossia research evaluation, patients also have tongue morphometric photographs captured. Those with symptomatic macroglossia are referred to the pediatric plastic surgeon, with approximately 25% of referred patients determined to be surgical candidates. Surgery is considered when patients have a primary indication of breathing and feeding concerns (typically in younger patients) or craniodental development concerns (typically in those older than 1 year of age). Comparing the morphometric measurements between patients who had surgery to those who did not, we found that tongues were more ridged in individuals with a surgical history. Ridging, which is primarily a measure of posterior lingual crowding, is likely unchanged pre- and postoperatively given that the posterior tongue is not surgically excised. Therefore, the finding of an association between increased ridging and surgical selection may be useful information for surgeons considering intervention. Specifically, if minimal ridging is present, then surgeons may be less inclined to consider surgical intervention, and vice versa.

There are several limitations to this study. While analysis of morphometric measurements revealed statistically significant findings, it is important to note that the limited sample size within these groups may impact the broader applicability of the results. Nevertheless, the Benjamini-Hochberg correction was employed to reduce the likelihood of type 1 errors in multiple comparison testing. Additionally, sleep studies and morphometric measurements were conducted at different times throughout patients’ treatment courses, but when we controlled for age between polysomnography and morphometric measurements, there was minimal change in outcomes. Further, we used morphometric measurement age when conducting statistical analysis. Lastly, we do not currently have pre- and post-tongue reduction morphometric data on patients, and this will be an area of future study.

## Conclusion

Objective intraoral morphometric measurements are associated with phenotypic burden in BWS. Tongue morphology varies across the BWS spectrum, with IC2 LOM presenting with wider tongues and mouths, and pUPD11 having narrower mouths. Tongue ridging is more common in those selected for surgery. These measurements are easy to obtain and may be adopted at low costs across centers caring for children with BWS or others at risk of upper airway obstruction.

## Electronic supplementary material

Below is the link to the electronic supplementary material.

Supplementary Material 1

## Data Availability

The data that support the findings of this study are not openly available due to reasons of sensitivity and are available from the corresponding author upon reasonable request. Data are located in controlled access data storage at the Children’s Hospital of Philadelphia.

## Abbreviations

BWS: Beckwith-Wiedemann Syndrome
IC2 LOM: Loss of Methylation at Imprinting Control Region 2
IC1 GOM: Gain of Methylation at Imprinting Control Region 1
pUPD11: paternal Uniparental Isodisomy of Chromosome 11p15
GWpUPD: Genome Wide paternal Uniparental Isodisomy
OSA: Obstructive Sleep Apnea
SpO_2_: Oxygen Saturation nadir
AHI: Apnea-Hypopnea Index
BIG: BWS Index of macroGlossia
OMNP: Open Mouth No Phonation
TEMA: Tongue Extended Maximally Anterior
TEML: Tongue Extended Maximally Lateral
MArea: Mouth Area
MWidth: Mouth Width
MHeight: Mouth Height
TArea: Tongue Area
TWidth: Tongue Width
TLength: Tongue Length
TCurve: Tongue Curvature
TThick: Tongue Thickness
