# Supplementary material for: Morphometric measurements of intraoral anatomy in children with Beckwith-Wiedemann syndrome: a novel approach
Source: Orphanet J Rare Dis. 2024 Oct 17;19:384. doi: 10.1186/s13023-024-03350-3 (PMC11483972; doi:10.1186/s13023-024-03350-3)
Supplement: Supplementary file 1 — Supplementary Material 1 [file 13023_2024_3350_MOESM1_ESM.docx]

| Supplementary Table 1. Percent Mosaicism Calculations | |
| --- | --- |
| IC2 LOM: | \| 0.5 – (IC2 methylation fraction) \| x 2 |
| IC1 GOM: | \| 0.5 – (IC1 methylation fraction) \| x 2 |
| pUPD11: | \| 0.5 – (IC2 methylation fraction) \| + \| 0.5 – (IC1 methylation fraction) \| |
